# Poly(2,6-Dimethyl-1,4-Phenylene Oxide)-Based Hydroxide Exchange Separator Membranes for Zinc–Air Battery

**DOI:** 10.3390/ijms20153678

**Published:** 2019-07-26

**Authors:** Ali Abbasi, Soraya Hosseini, Anongnat Somwangthanaroj, Ahmad Azmin Mohamad, Soorathep Kheawhom

**Affiliations:** 1 Department of Chemical Engineering, Faculty of Engineering, Chulalongkorn University, Bangkok 10330, Thailand; 2 Computational Process Engineering Research Laboratory, Chulalongkorn University, Bangkok 10330, Thailand; 3 School of Materials and Mineral Resources Engineering, Universiti of Sains Malaysia, Nibong Tebal, Pulau Pinang 14300, Malaysia

**Keywords:** zinc–air battery, separator, hydroxide exchange membrane, anion-exchange membrane, ionic channel, polyphenylene oxide

## Abstract

Rechargeable zinc–air batteries are deemed as the most feasible alternative to replace lithium–ion batteries in various applications. Among battery components, separators play a crucial role in the commercial realization of rechargeable zinc–air batteries, especially from the viewpoint of preventing zincate (Zn(OH)_4_^2−^) ion crossover from the zinc anode to the air cathode. In this study, a new hydroxide exchange membrane for zinc–air batteries was synthesized using poly (2,6-dimethyl-1,4-phenylene oxide) (PPO) as the base polymer. PPO was quaternized using three tertiary amines, including trimethylamine (TMA), 1-methylpyrolidine (MPY), and 1-methylimidazole (MIM), and casted into separator films. The successful synthesis process was confirmed by proton nuclear magnetic resonance and Fourier-transform infrared spectroscopy, while their thermal stability was examined using thermogravimetric analysis. Besides, their water/electrolyte absorption capacity and dimensional change, induced by the electrolyte uptake, were studied. Ionic conductivity of PPO–TMA, PPO–MPY, and PPO–MIM was determined using electrochemical impedance spectroscopy to be 0.17, 0.16, and 0.003 mS/cm, respectively. Zincate crossover evaluation tests revealed very low zincate diffusion coefficient of 1.13 × 10^−8^, and 0.28 × 10^−8^ cm^2^/min for PPO–TMA, and PPO–MPY, respectively. Moreover, galvanostatic discharge performance of the primary batteries assembled using PPO–TMA and PPO–MPY as initial battery tests showed a high specific discharge capacity and specific power of ~800 mAh/g_Zn_ and 1000 mWh/g_Zn_, respectively. Low zincate crossover and high discharge capacity of these separator membranes makes them potential materials to be used in zinc–air batteries.

## 1. Introduction

Recently, metal–air batteries have attracted high interest of researchers and industry as post lithium–ion technology. Among all metal–air batteries, aqueous zinc–air battery is a relatively established technology (known to the scientific community since the late nineteenth century) with high potential to be used in future energy requirements [1,2,3]. This type of battery has a very high theoretical energy density of 1086 Wh·kg^−1^ (including oxygen), five times higher than that of existing lithium–ion batteries [4]. Moreover, their production cost is estimated to be very low (~10 $·kW^−1^·h^−1^) in comparison to lithium–ion batteries. Technically and economically, the zinc–air battery is deemed to be the most feasible alternative for lithium–ion batteries in various applications. Despite the early start and great potential, the development of zinc–air batteries has been limited by issues related to separator, electrolyte, metal electrode, and air catalyst [5].

Despite being an essential part of a zinc–air battery, the separator has not received its deserved attention compared to other components of the battery. The primary function of the separator is to prevent physical contact between the anode and cathode, while providing high ionic conductivity and selectivity to facilitate hydroxide ion transport with the aim of completing the battery circuit during its operation. An ideal separator must be chemically stable in contact with highly alkaline environment and electrochemically stable within a wide working potential window. Also it must be able to prevent zincate (Zn(OH)_4_^2−^) crossover and short-circuit due to the formation of zinc dendrites during battery charging. Besides, high electrical resistance and ionic conductivity is highly desirable [6].

Practically, the separator for zinc–air batteries is usually a porous polypropylene membrane such as Celgard^®^ with the porosity of 10–20 µm. The porosity of these membranes results in their high ionic conductivity, which is crucial for a separator material. Nonetheless, due to the high porosity, beside hydroxide ions, zincate ions, which are produced through the oxidation of zinc during the battery discharge, also pass through the membrane from the anode to the cathode [7]. At the cathode side, because of asymmetric water evaporation-induced electrolyte deficiency, zincate ions are precipitated as zinc oxide (ZnO) on the catalyst surface, forming a resistive layer to ion/electron conduction which in turn, leads to higher polarization of air electrode and capacity loss of zinc–air batteries [8].

Even though there have been several attempts to develop a separator membrane with desired characteristics to be used in zinc–air batteries, more efforts are required in this field. Dewi et al. used a polyelectrolyte containing a sulfonium cation as a zinc–air battery separator. Even though they incorrectly assumed zinc ions in the electrolyte to be in the form of a cation (Zn^2+^), the separator was highly effective in preventing zinc ions crossover to the cathode side, leading to the capacity increase by more than six times compared to polypropylene-based Celgard^®^ separator [7]. More recently, electrospun polyetherimide (PEI) nanofibers impregnated with polyvinyl alcohol (PVA) has been used as a highly efficient separator for rechargeable zinc–air battery. In the prepared permselective membrane, PEI, which is known for its high chemical resistance in the alkaline environment, provides good mechanical stability. On the other hand, the pores of the electrospun nanofibers filled with PVA electrolyte offers high ionic conductivity. In such a design, zincate crossover decreased dramatically due to the bulky size of the zincate ions, making it difficult to pass through the separator compared to hydroxide ions [8]. In another study, similar impregnated nanofibrous mat concept with electrospun PVA/polyacrylic acid (PAA) nanofibers along with the impregnation solution of Nafion bearing pendant sulfonate groups was used. In this case, the nanofibers acted as the ionic conductive pathways for hydroxide ions, and the impregnation solution played the role of an anion repelling component, preventing bulky zincate ions transport from the anode to the cathode side [9].

Basically, the ionic conductivity of polymeric materials is determined by two factors: the ionic mobility and the ion exchange capacity (IEC). To increase hydroxide conductivity, improving IEC seems to be an easier way than improving hydroxide mobility. However, increasing IEC always results in excessive water uptake, which is a negative side effect, leading to severe swelling or even dissolution at higher temperatures. Moreover, increasing IEC usually leads to an increased zincate crossover. Therefore, a better and more efficient approach to expanding the hydroxide conductivity and selectivity is to enhance hydroxide mobility while keeping the IEC at a medium level. Such a growth in the hydroxide conductivity could be realized by reforming the ionic channels in polymer structure through hydrophilic/hydrophobic microphase separation. In this approach, a cation which is usually quaternary ammonium (–NR_3_^+^), is covalently bound to a hydrophobic polymer backbone and the anion (hydroxide) is dissociated in aqueous phase [10]. Several polymeric materials have been used to develop this type of separator membrane such as poly(arylene ether)s, poly-(phenylene)s, poly(ether imide)s, poly(styrene)s, poly(olefin)s, and poly(phenylene oxide)s [11].

Recently, there has been high interest in poly (phenylene oxide) (PPO) as the polymer backbone. PPO exhibits low-cost; commercial availability; high thermal, mechanical, and chemical stabilities; and facile postfunctionalization [12]. Li et al. designed and synthesized comb-shaped quaternized copolymer using PPO as the base polymer. The synthesis was carried out using Menshutkin reaction with N,N-dimethyl-1-hexadecylamine (DMHDA), and subsequent hydroxide exchange. In this process, DMHDA was attached to the benzylic position of PPO through covalent bonding from nitrogen atom, forming a quaternary ammonium group directly attached to the polymeric backbone with a pendant long hydrocarbon chain. The polymer exhibited phase-separated morphology with enhanced ionic conductivity and alkaline stability, making it a potential material as hydroxide exchange membrane for alkaline fuel cells [13]. In another study, Yang et al. prepared various quaternized PPO-based anion-exchange membranes using a series of saturated heterocyclic compounds including 1-methylpyrrolidine (MPy), 1-ethylpyrrolidine (EPy), 1-butylpyrrolidine (BPy), 1-methylpiperidine (MPrD), 1-ethylpiperidine (EPrD), and N-methylmorpholine (NMM). Also, the comparison of their physicochemical characteristics with TMA-quaternized PPO as the benchmark polymer was performed. The quaternized membranes showed different ionic conductivity and alkaline stability, depending on the quaternization agent used. For example, the polymer containing 1-methylpyrolidine exhibited ionic conductivity of 27 mS·cm^−1^ at 80 °C and excellent alkaline stability, keeping 87% of its original conductivity after being soaked in 1 M KOH at 80 °C for 500 h [14].

Although this strategy has already been used in fuel cell technology, its application in zinc–air batteries, where an alkaline stable hydroxide exchange membrane is required as well, is very rare. The main focus of this study is to synthesize PPO-based microphase separated hydroxide exchange membrane through the attachment of three various quaternary ammonium molecules to the main polymeric backbone with the aim of developing ionic channels for selective transfer of hydroxide ions. Even though the main aim of developing new separators with acceptable ionic conductivity and low zincate crossover is for overcoming challenges associated with rechargeable zinc–air batteries, this phase of our study includes separators characterization and their application in a primary zinc–air battery as an indication of their applicability in secondary batteries. The separators are characterized using physicochemical and electrochemical characterization tools to investigate their properties, including their ionic conductivity and zincate diffusion coefficient. Then, they are implemented in a primary zinc–air battery to study their influence on battery performance. The ionic conductivity, along with suppressed zincate crossover, makes this new separator an excellent candidate to be used in primary and secondary zinc–air batteries. In the next phase, the application of the separators in rechargeable zinc–air batteries will be investigated.

## 2. Results and Discussion

### 2.1. Separator Membrane Synthesis

**Figure 1:**
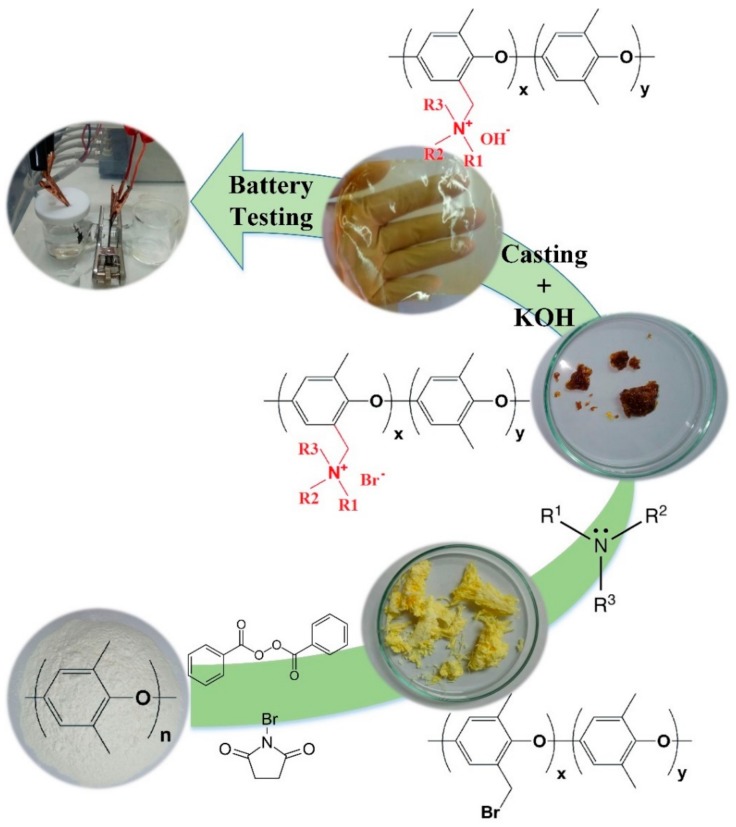
Schematic diagram of the membrane preparation process.

Schematic view of the membrane synthesis process is illustrated in Figure 1. In the first stage, a mixture of N-bromosuccinimide (NBS) and benzoyl peroxide (BPO) is used for the bromination reaction. NBS acts as the bromination agent, and BPO is the initiator of the reaction. Controlling the temperature and reaction condition is very crucial to ensure that the bromination reaction takes place dominantly in the benzylic position and aromatic hydrogen atoms are not being involved in the reaction. Quaternization using three amine molecules including trimethylamine (TMA), 1-methylpyrrolidine (MPY), and 1-methylimidazole (MIM) was carried out at room temperature in 48 h using an excessive amount of the amine molecules to make sure of complete reaction. Subsequently, the quaternized polymer was casted to fabricate uniform and flexible films with the thickness of 40 to 60 µm. In the final stage, the films were soaked in potassium hydroxide (KOH), 7 M solution for 72 h for completing hydroxide exchange of bromine ions in the polymer structure. To make sure of the ion exchange process, every 24 h, the hydroxylation solution was changed to a fresh solution [15]. Three PPO-based separator membranes containing trimethylamine (PPO–TMA), 1-Methylpyrrolidine (PPO–MPY) and 1-methylimidazole (PPO–MIM) were synthesized.

### 2.2. Structural Changes

#### 2.2.1. Proton Nuclear Magnetic Resonance

**Figure 2:**
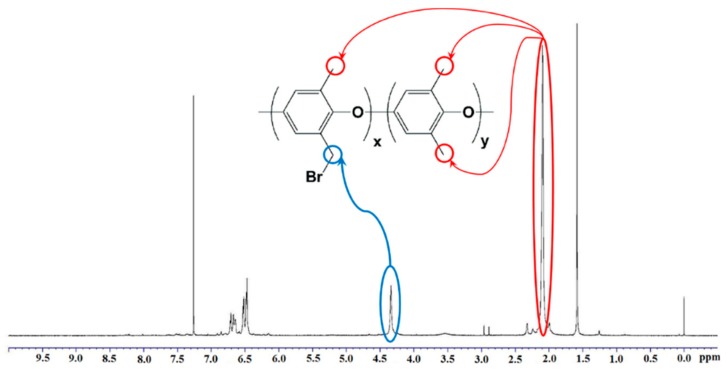
^1^HNMR spectra of BrPPO in CDCl_3_. The blue line indicates the characteristic peak of brominated methyl protons (–CH_2_Br). The red line indicates the characteristic peak of methyl (benzylic: –CH_3_).

Figure 2 shows the proton nuclear magnetic resonance (^1^HNMR) spectrum of the brominated PPO. As previously reported in [16], pure PPO shows two characteristic peaks at around 2.1 and 6.5 ppm, assigned to the methyl (benzylic: –CH_3_) and aryl protons, respectively. After bromination, a new peak appeared at 4.3 ppm, which is attributed to the brominated methyl protons (–CH_2_Br). Furthermore, no peak was observed at 6 ppm (assigned to shifted aryl protons due to the bromination of neighboring aryl protons), showing that the bromination occurred dominantly at benzylic positions [17]. Considering the ratio of the integral area of CH_3_ and CH_2_, the bromination degree of the PPO was calculated to be 39%. Successful bromination reaction was confirmed using ^1^HNMR spectrum [14].

#### 2.2.2. Fourier-Transform Infrared Spectroscopy

**Figure 3:**
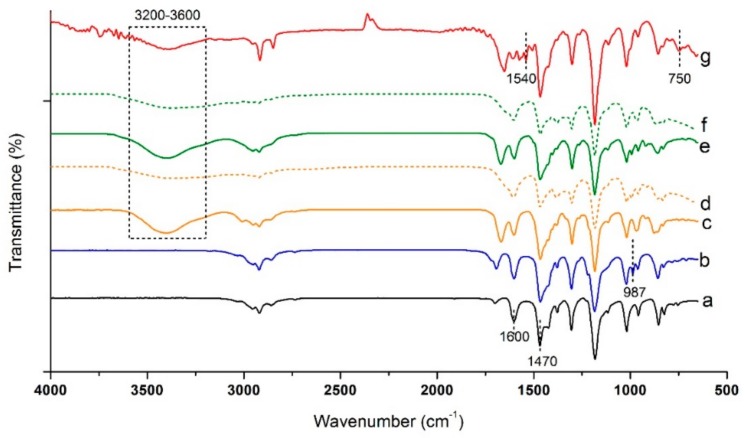
FTIR spectra of (**a**) PPO, (**b**) BrPPO, (**c**) PPO–TMA, (**d**) PPO–TMA after 150 h soaking in KOH, 7 M (**e**) PPO–MPY, and (**f**) PPO–MPY after 150 h soaking in KOH, 7 M, and (**g**) PPO–MIM. The broad peak shown in the dash line frame is attributed to O–H stretching of the water molecules absorbed into the samples due to their increased hydrophilicity after quaternization.

A successful synthesis of BrPPO and associated quaternized membranes was also confirmed by Fourier-transform infrared (FTIR) spectroscopy, as shown in Figure 3. Characteristic peaks of phenyl group of PPO appeared at about 1600 and 1470 cm^−1^, corresponding to C=C stretching of the benzene ring and C–H stretching, respectively [14,16]. Compared to PPO, a new peak appeared at about 987 cm^−1^ for BrPPO, which was assigned to C-Br stretching [18]. After quaternization using TMA, MPY, and MIM, C-Br peak disappeared, approving complete quaternization reaction. Furthermore, for all quaternized samples, a broad peak appeared at 3200–3600 cm^−1^, attributed to O–H stretching of the water molecules absorbed into the samples due to their increased hydrophilicity after quaternization. Moreover, for PPO–MIM, two strong absorption peaks appeared at about 750 and 1540 cm^−1^, related to the presence of imidazolium cations in the sample [19]. FTIR results also show successful bromination of PPO and nucleophilic substitution of BrPPO with quaternization agents.

Furthermore, to have an initial evaluation of the separator membranes’ chemical stability, PPO–TMA and PPO–MPY were soaked in KOH, 7 M solution for 150 h at 30 °C, and then, after washing and drying, they were analyzed using FTIR. As could be seen in Figure 3d,f, they showed similar characteristic peaks after soaking in the alkaline solution, and no significant change was observed in their spectrum, showing their stability in the solution used for zinc–air batteries.

### 2.3. Thermal Properties

**Figure 4:**
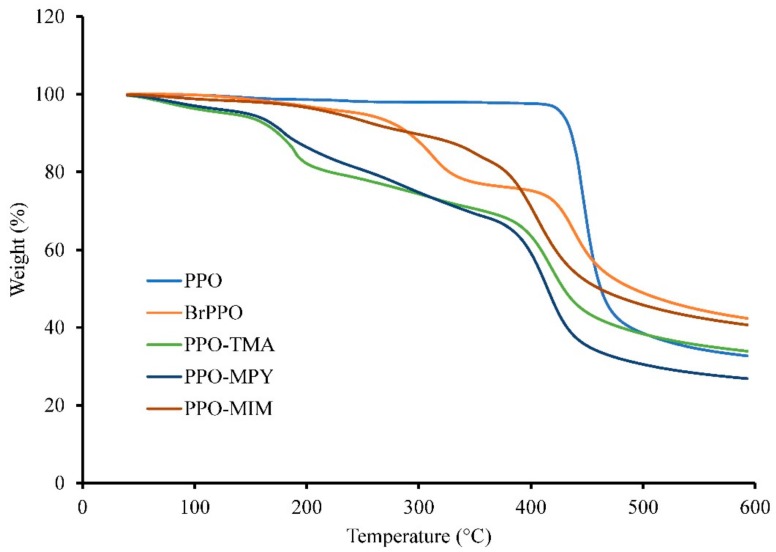
TGA curves of PPO, BrPPO, PPO–TMA, PPO–MPY, and PPO–MIM.

Figure 4 shows thermogravimetric analysis (TGA) curves for various separator membranes in their hydroxide form. Pristine PPO shows a single degradation step at 438 °C, as studied before [14]. The decomposition of BrPPO happened in two steps, with the first related to the degradation of brominated parts at 312 °C and the second for the PPO backbone decomposition at 438 °C [20]. Furthermore, all quaternized samples showed a three-step similar degradation behavior. The first step at approximately 90 to 150 °C was ascribed to the removal of moisture absorbed onto hydrophilic samples. The intensity of this peak for PPO–MIM was lower than those of PPO–TMA and PPO–MPY, showing its lower hydrophilicity, as confirmed by its lower water uptake (Table 1). The second peak at ~180–250 °C was attributed to the degradation of various quaternary ammonium groups, and the final degradation step was for PPO backbone at 405–420 °C [14]. Second degradation peak of PPO–MIM, related to the decomposition of 1-methylimidazolium quaternary ammonium group, occurred at a higher temperature compared to PPO–TMA and PPO–MPY. This could be attributed to the presence of an aromatic ring in its structure, leading to higher thermal stability [21]. TGA results revealed that all PPO-based separator membranes meet the thermal stability requirement for being used in zinc–air battery applications, which are usually operated at room temperature, or at the highest temperature below 80 °C [5].

### 2.4. Electrochemical Characterization

#### 2.4.1. Ionic Conductivity

Separator membranes need to absorb water/electrolyte to be able to conduct ions. Table 1 shows water and electrolyte uptake for various PPO-based separators along with their area and volume change induced by the uptake. As could be seen, PPO–TMA and PPO–MPy show high water uptake of 89 and 78%, respectively, showing their high hydrophilicity. However, the water uptake of PPO–MIM was as low as 13%, resulting from its low tendency to water absorption. This water uptake led to corresponding area and volume increase in the membranes, as shown in the table. Similar results have been obtained in previous studies for these three membranes [14].

**Figure 5:**
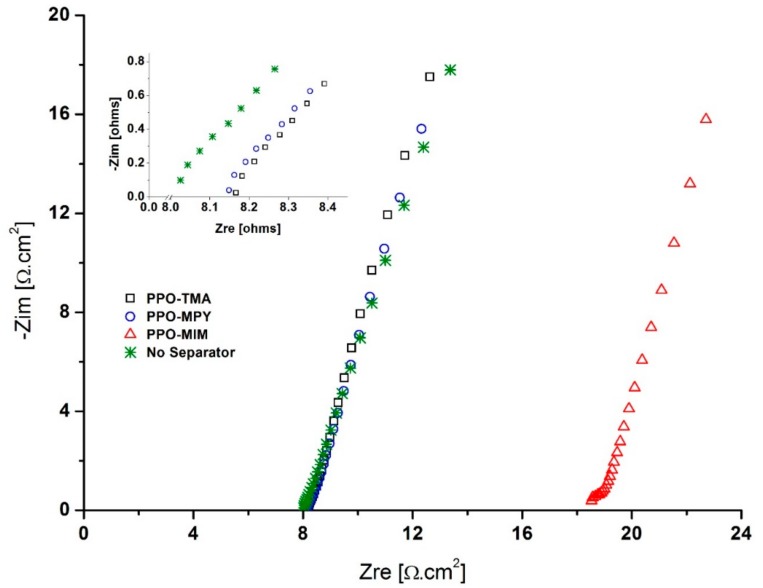
Nyquist plot of electrochemical impedance spectroscopy (EIS) for determining ionic conductivity of PPO-based separator membranes.

Since in the zinc–air cell KOH, 7 M is used as the electrolyte, the uptake and dimensional change of the membranes were also measured in this electrolyte. All the membranes absorbed less electrolyte compared to water (~30% for PPO–TMA and PPO–MPY, and only 3% for PPO–MIM), leading to lower dimensional change. Also, very low electrolyte uptake of PPO–MIM was reflected in the ionic conductivity measurements, showing very low conductivity of 0.003 mS/cm determined using Nyquist plot of electrochemical impedance spectroscopy (EIS) (Figure 5). For PPO–TMA and PPO–MPy, the ionic conductivity was calculated to be 0.17 and 0.16 mS/cm. Due to deficient electrolyte uptake and low ionic conductivity of PPO–MIM, it was not included in the rest of the study.

Interestingly, much higher ionic conductivities have been reported for the same separator membranes [14]. However, it should be noted that usually the ionic conductivity is measured in water, leading to higher water uptake by the separator and as a result, higher ionic conductivity. In this study, the measurements were carried out in KOH, 7 M solution to mimic the real cell operation condition. As could be seen in Table 1, the separator membranes absorb much less electrolyte than they do in water, resulting in lower measured ionic conductivity.

#### 2.4.2. Zincate Crossover

To realize electrochemically rechargeable Zn–air batteries, minimizing zincate crossover from the anode to the cathode is essential. In this regard, highly selective separator materials with acceptable hydroxide conductivity and limited zincate ion conductivity play an important role. In this study, a two-chamber diffusion cell was used to quantitatively investigate the zincate crossover through the developed separator membranes. The left chamber contained KOH, 7 M solution plus 0.5 M dissolved ZnO in the form of zincate ions and the right chamber contained only KOH, 7 M solution. The separator membrane was place between two chambers and the concentration gradient-driven crossover of zincate ions to the right chamber was measured as a function of time using inductively coupled plasma atomic emission spectroscopy (ICP–OES).

**Figure 6:**
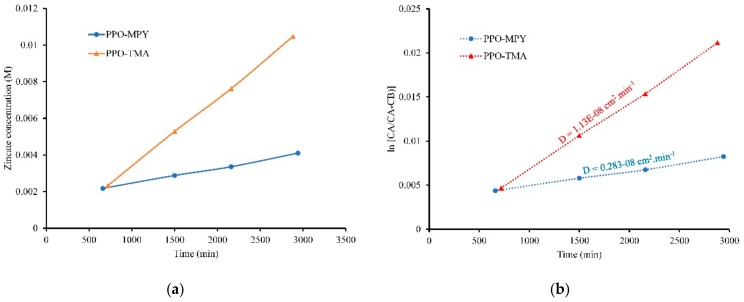
Variation of the zincate concentration in the right chamber vs. time (**a**) and zincate diffusion calculation curves (**b**) for PPO–TMA and PPO–MPY separators.

As could be seen in Figure 6a, the concentration of zinc ions in the right chamber increased with time for both PPO–TMA and PPO–MPY separator membranes. However, the slope of increase for PPO–MPY was lower than that of PPO–TMA, showing its lower zincate crossover during time. Furthermore, the diffusion coefficient of zincate ions through the separators was calculated using Equation 5. Besides, Figure 6b depicts the diffusion coefficient for two separator membranes. As can be seen, the diffusion coefficient for PPO–TMA and PPO–MPY was 1.13 × 10^−8^ and 0.28 × 10^−8^ cm^2^/min, respectively, indicating lower zincate crossover for PPO–MPY compared to PPO–TMA. Nevertheless, in comparison with other separators previously studied, such as Celgard3501 (232.4 × 10^−7^ cm^2^/min), PVA/PAA film (110.2 × 10^−7^ cm^2^/min), Nafion bearing electrospun PVA/PAA mat (4.1 × 10^−7^ cm^2^/min) [9] PEI nanofibers impregnated with PVA (5.0 × 10^−6^ cm^2^/min) [8], and even Nafion (0.4 × 10^−7^ cm^2^/min) [9]; PPO-based membranes have much lower zincate diffusion coefficient, making them highly desirable for zinc–air battery separators. This could be ascribed to the formation of ionic channels in the polymer structure through hydrophilic/hydrophobic microphase separation. As a result, the crossover of bulky zincate ions through the membrane separator was suppressed, but smaller hydroxide ions could pass through the separator.

#### 2.4.3. Electrochemical Stability

**Figure 7:**
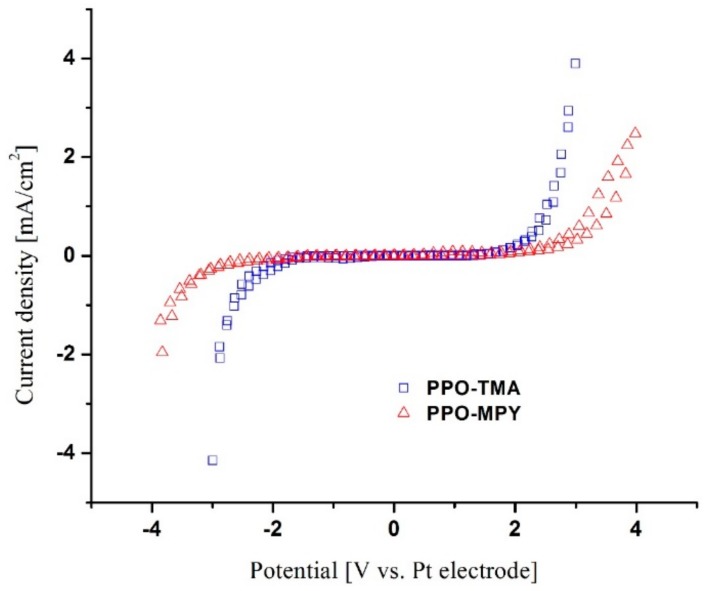
Cyclic voltammograms of PPO–TMA and PPO–MPY.

The electrochemical stability window, which is defined as the width of voltage where no appreciable faradaic current flows, could be studied using cyclic voltammetry (CV). A wide electrochemical window is very crucial for the application of the membranes in batteries [22]. As could be seen in Figure 7, for PPO–TMA, there was no significant decomposition of separator components observed in a range of −1.5 to +1.5 V, providing a stability widow of 3 V. For PPO–MPy, the stability window was measured to be 4 V, which is even higher than that of PPO–TMA. These extensive electrochemical stability windows, wider than most of the previously studied separator materials [8,9,22,23], make PPO-based separator membranes a highly stable candidate to be used in battery applications, without any worry about the membranes themselves being involved in the electrochemical reaction during charge and discharge cycles.

### 2.5. Discharge Performance

Zincate crossover affects the zinc–air battery performance in two main ways: (1) high crossover of zincate ions makes zinc ions inaccessible for the anode electrode during the charging process, negatively affecting battery performance. This is important for electrically rechargeable zinc–air batteries. (2) Due to electrolyte deficiency at the cathode side, zincate ions are precipitated as zinc oxide on the surface of catalyst particles, resulting in higher polarization of air electrode and capacity loss of zinc–air batteries because of formation of a resistive layer to ion/electron conduction. This will affect both primary and secondary zinc–air batteries [8]. Even though the main purpose of developing new separator material with low zincate crossover is to overcome the challenges associated with electrically rechargeable zinc–air batteries, in this study the PPO-based separators were used in a primary battery to have an indication of its applicability in secondary batteries, which will be the next stage of our work.

**Figure 8:**
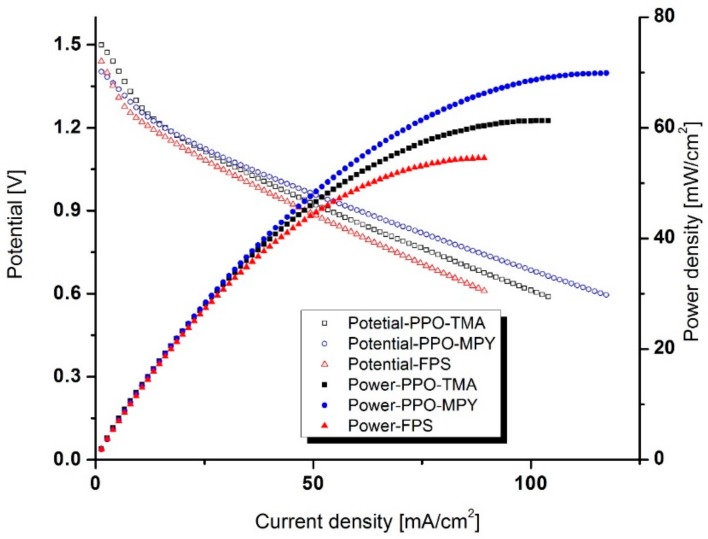
Polarization characteristics of the cells with various separators of PPO–MPY, PPO–TMA, and filter paper-based separator (FPS).

The prepared separator membranes were integrated into a homemade zinc–air battery with effective separator and cathode area of 1.77 cm^2^. Figure 8 shows the polarization characteristics of pristine zinc plate in KOH, 7 M in a zinc–air cell using various separator membranes. The voltage and power of the cells show a strong dependency on the discharge current. Except for the initial sharp drop, all the cells showed a linear voltage decrease with an increase in the discharge current, revealing the dominance of ohmic losses on the cell performances [24]. Filter paper-based separator (FPS) was used as a benchmark separator. The intensity of ohmic losses for various separator membranes were in the order of FPS > PPO–TMA > PPO–MPY, revealing their ohmic resistance in the cell operation condition. The cells with FPS, PPO–TMA, and PPO–MPY showed maximum discharge current density of 89, 104, and 117 mA/cm^2^ with a maximum power density of 55, 61, and 70 mW/cm^2^, respectively. The comparison demonstrates lower ohmic loss for PPO-based membranes compared to FPS as a benchmark separator. It could be attributed to the formation of ionic channels in the structure of the membranes, facilitating hydroxide ions traveling through the separator.

**Figure 9:**
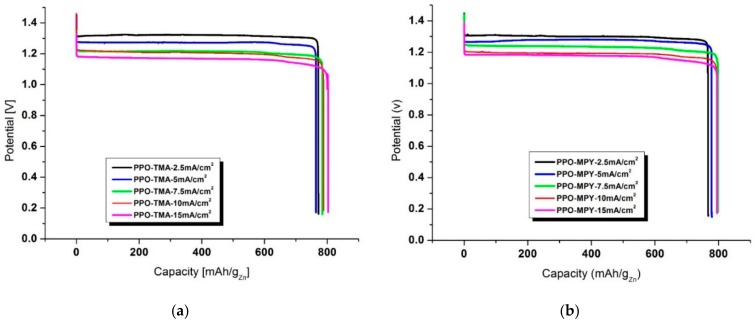
Galvanostatic discharge profile of the cell with PPO–TMA (**a**) and PPO–MPY (**b**) as a separator membrane at discharge current densities of 2.5 to 15 mA/cm^2^.

Galvanostatic discharge profiles of zinc–air cells assembled using PPO–TMA and PPO–MPY separator membranes at discharge current densities in the range of 2.5 to 15 mA/cm^2^ are presented in Figure 9. For PPO–TMA (Figure 9a), the highest discharge capacity and power of 803 mAh/g_Zn_ and 932 mWh/g_Zn_ was obtained for discharge current of 15 mA/cm^2^ at 0.9 V cut-off voltage. With decreasing the discharge current density, discharge time increased, and discharge capacity decreased to ~770 mAh/g_Zn_ for 2.5 mA/cm^2^. This could be attributed to the higher hydrogen evolution and zinc corrosion reaction due to the elongated cell operation time [24]. However, because of having higher discharge voltage at lower discharge current densities (lower ohmic loss), the power increased to ~1015 mWh/g_Zn_ for 2.5 mA/cm^2^ discharge current density. A similar trend was observed for the cell using PPO–MPY as a separator membrane, as could be seen in Figure 9b. The highest specific capacity of 795 mAh/g_Zn_ was achieved at a discharge current density of 15 mA/cm^2^, decreasing to 772 mAh/g_Zn_ for 2.5 mA/cm^2^. The power of the cell increased from 931 mWh/g_Zn_ for 15 mA/cm^2^ to 996 mWh/g_Zn_ for 2.5 mA/cm^2^ discharge current density. The cells assembled using both PPO-based separator membranes exhibited very high specific capacity and power [8].

**Figure 10:**
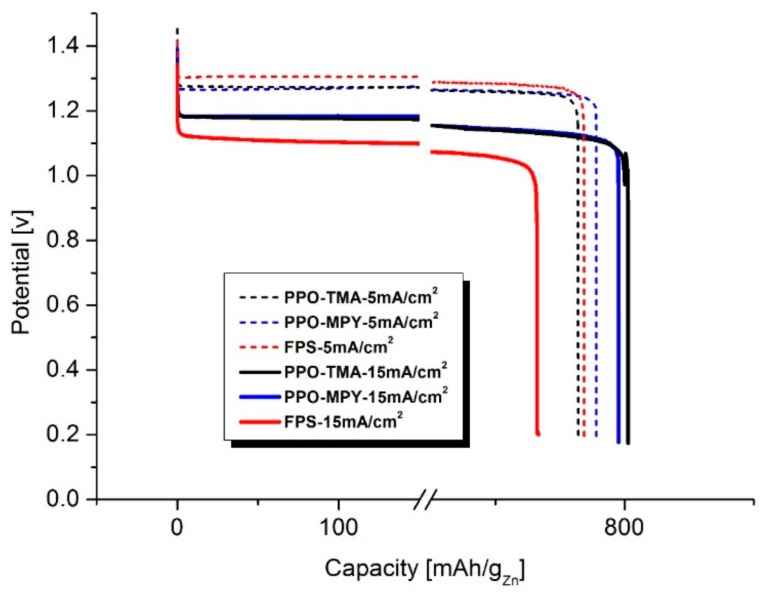
Galvanostatic discharge profile of the cells with PPO–TMA, PPO–MPY, and FPS as separator membrane at discharge current densities of 5 and 15 mA/cm^2^.

A comparison between the specific capacity of the cells assembled using PPO–TMA, PPO–MPY, and FPS at discharge current densities of 5 and 15 mA/cm^2^ is shown in Figure 10. As could be seen, for 5 mA/cm^2^, the capacity of the cells is in the same range of ~770 mAh/g_Zn_ for all three membranes. However, with increasing the discharge current density to 15 mA/cm^2^, the capacity of the cells using PPO-based membranes increases to around 800 mAh/g_Zn_ due to shorter discharge time and so, lower hydrogen evolution reaction. Nonetheless, for FPS containing cell, the capacity decreased to ~730 mAh/g_Zn_. It reveals the dominance of ohmic losses for this separator membrane in high discharge current densities. These results are in agreement with the polarization curve of PPO-based membranes and FPS, showing a lower ohmic loss for PPO-based separators.

## 3. Materials and Methods

### 3.1. Materials

Poly(2,6-dimethyl-1,4-phenylene oxide) powder (PPO), 1-methylpyrrolidine (≥98%), and manganese (IV) oxide (5 μm, 99.99%) were supplied by Sigma-Aldrich (St. Louis, MO, USA). *N*-bromosuccinimide (NBS) for synthesis, benzoyl peroxide (BPO, with 25% H_2_O), and chlorobenzene were purchased from Merck Millipore. 1-methyl-2-pyrrolidinone (NMP), ethanol, methanol, and toluene all with Grade AR were purchased from QRëC (New Zealand). *N*,*N*-dimethylformamide (DMF) (LOBA Chemie, Grade AR, Mumbai, India), trimethylamine (TCI, ca. 25% in methanol), 1-methylimidazole (TCI, ≥99%), chloroform (BDH Chemicals, UK), and KOH plates (Kemaus, Australia) were used as received without further purification. Nickel (Ni) foam as cathode current collector with a purity of 99.97%, 100 pores per inch (PPI), and 1 mm thick was purchased from Qijing Trading Co., Ltd. (Wenzhou, China). Carbon black (Vulcan^®^ XC-72, Cabot Corporation, Boston, MA, USA), BP-2000 (BLACK PEARLS^®^ 2000, Cabot Corporation), and zinc plate (0.1 mm thick 99.99%, Shandong Yr Electronic Co., Ltd., Shandong, China) were used as received. Poly (styrene-co-butadiene) (Sigma-Aldrich, butadiene 4 wt%) was used to prepare binder for the cathode. Poly(vinyl acetate) (PVAc) from TOA Paint Public Co., Ltd. (Samut Prakarn, Thailand) and No. 4 Whatman filter paper (Sigma-Aldrich) were used to prepare the benchmark separator.

### 3.2. Separator Membrane Synthesis

PPO-based phase-separated hydroxide exchange separator membranes were synthesized using a process described previously [19]. The first stage of separator preparation was bromination of PPO at the benzylic position. A certain amount of PPO was dissolved in chlorobenzene at 50 °C under nitrogen atmosphere to prepare 5 wt/*v*% solution. Then, the temperature was increased to 80 °C, NBS (with PPO:NBS weight ratio of 1:0.8) and BPO (with NBS:BPO weight ratio of 1:0.05) were added to the mixture, and the reaction continued for 4 h under nitrogen atmosphere. The solution was then cooled down to room temperature and poured into an excessive amount of methanol for product precipitation. A pale-yellow fiber-like product was separated and then dissolved in chloroform for purification. The solution was precipitated again in an excessive amount of ethanol, filtered and dried in the vacuum oven at 60 °C overnight.

The next stage was to introduce quaternary ammonium functional group. Brominated PPO (BrPPO) was dissolved in NMP with the concentration of 7.5 wt/*v*%, and then an excessive amount of the quaternization agent (trimethylamine, 1-methylpyrrolidine, and 1-methylimidazole) was added to the solution. The reaction continued for 48 h in room temperature, and then the product was precipitated using toluene, washed a few times and then dried in a vacuum oven at 65 °C for 24 h [14].

In the next stage, a certain amount of quaternized BrPPO (Q-BrPPO) was dissolved in DMF at room temperature to prepare 30 wt/*v*% casting solution. The membrane was fabricated by casting the prepared solution onto a clean surface. Finally, the prepared cast films were soaked in degassed KOH, 7 M solution for 72 h to exchange the bromine ions in the polymer structure with hydroxide. Fresh hydroxide exchange solutions were used every 24 h to make sure of complete ion exchange [15].

To evaluate the performance of the synthesized separator membranes, a benchmark membrane using Whatman filter paper was also prepared. For this purpose, both sides of No.4 Whatman filter paper was coated with a 24 wt% PVAc solution and dried in an oven at 60 °C for 15 min.

### 3.3. Structural/Physicochemical Characterization

The ^1^HNMR spectrum of BrPPO was obtained on a Bruker, Avance III HD 500 MHz using deuterated trichloromethane (CDCl_3_) as a solvent to determine the bromination degree of PPO. A Perkin Elmer, Spectrum One Fourier-transform infrared (FTIR) spectrometer in the frequency range of 4000 to 600 cm^−1^ was carried out to study the chemical changes in the molecular structure during various synthesis stages. Thermogravimetric analysis was performed using SDT-Q600 TGA instrument in the temperature range of 30 to 600 °C at a heating rate of 10 °C min^−1^ to evaluate the thermal stability of the samples.

To measure water/electrolyte uptake capacity of the membranes, they were soaked in water/KOH, 7 M solution for 24 h and the weight differences before and after soaking were used for the measurements using Equation 1 [8].

Δ*W* (%) = [(*W*_wet_ – *W*_dry_)/*W*_dry_] × 100 (1)

where W_wet_ and W_dry_ are the weights of the membranes after and before soaking in water/electrolyte, respectively. Similarly, dimensional changes of the membranes induced by water/electrolyte uptake were calculated by Equations 2 and 3 [8].

Δ*A* (area-based) (%) = [(*A*_wet_ – *A*_dry_)/*A*_dry_] × 100 (2)

Δ*V* (volume-based) (%) = [(*V*_wet_ – *V*_dry_)/*A*_dry_] × 100 (3)

### 3.4. Electrochemical Characterization

The ionic conductivity of the prepared membranes was measured using a potentiostat/galvanostat with impedance measurement unit (AMETEK, PAR VersaSTAT 3A) in the frequency range of 1 Hz–100 kHz with the excitation voltage of 10 mV_RMS_ at room temperature. For this measurement, a diffusion cell with two chambers was used. EIS measurement with and without the membrane placed between two chambers containing KOH, 7 M was performed, and the difference in the bulk resistance (*R*_b_) of the two measurements was used to calculate the ionic conductivity of the membranes using Equation 4 [25].

σ = *l*/*R*_b_·*A* (4)

σ is the ionic conductivity (S/cm), *R*_b_ is the bulk resistance (Ω), and *l* and *A* are thickness (cm) and area (cm^2^) of the membrane, respectively.

Cyclic voltammetry (CV) was carried out using an AMETEK, PAR VersaSTAT 3A potentiostat/galvanostat to evaluate electrochemical stability window of the membranes. Two-electrode configuration tests using 1 × 1 cm^2^ platinum (Pt) working and counter electrodes were carried out for CV with a scan rate of 0.05 mV/s.

**Figure 11:**
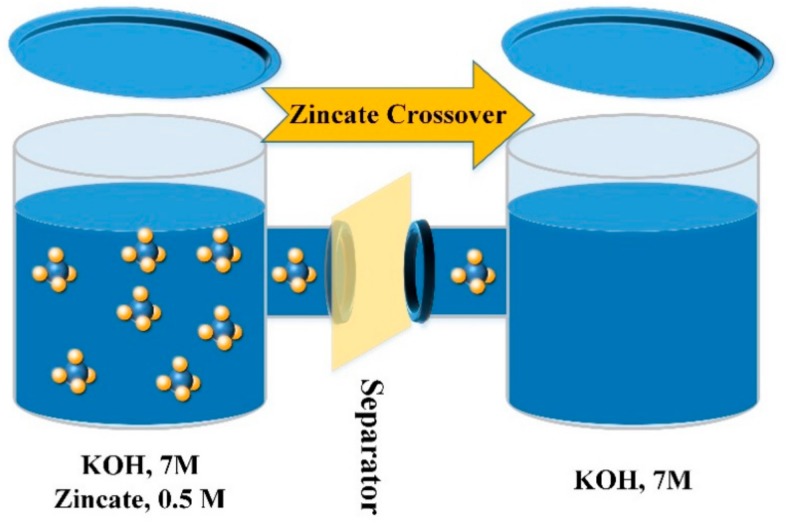
Schematic view of the diffusion cell used to measure zincate crossover.

To study zincate ion (Zn(OH)_4_^2-^) crossover characteristics of the membranes, a kind of diffusion cell with two chambers was used [9]. The left chamber contained 50 mL of KOH, 7 M solution plus 0.5 M dissolved ZnO in the form of zincate ions while the right chamber contained only 50 mL of KOH, 7 M solution (Figure 11). The separator membrane was placed between two chambers. The chambers were stirred continuously to prevent concentration polarization. The concentration of zinc ions in the right chamber was measured in a predetermined time interval (12–14 h) using inductively coupled plasma optical emission spectroscopy (ICP-OES) to obtain time-dependent concentration variation graph. Moreover, the diffusion coefficient of zincate ions across the separator membranes was calculated from the experimental data using Equation 5 [9].

ln(*C*_A_/(*C*_A_ − *C*_B_)) = (*D*·*A*/*V*_B_·*L*)·*t* (5)

*D* is the diffusion coefficient of zincate ions across the separator membrane (cm^2^/min), *t* is the time (min), *V*_B_ is the solution volume in the right chamber (deficiency chamber), *A* is the effective surface area (cm^2^) of the separator, *L* is the thickness (cm) of the separator, and *C*_A_ and *C*_B_ are the concentration of zincate ions (mol/L) in the left and right chambers, respectively.

### 3.5. Discharge Performance

**Figure 12:**
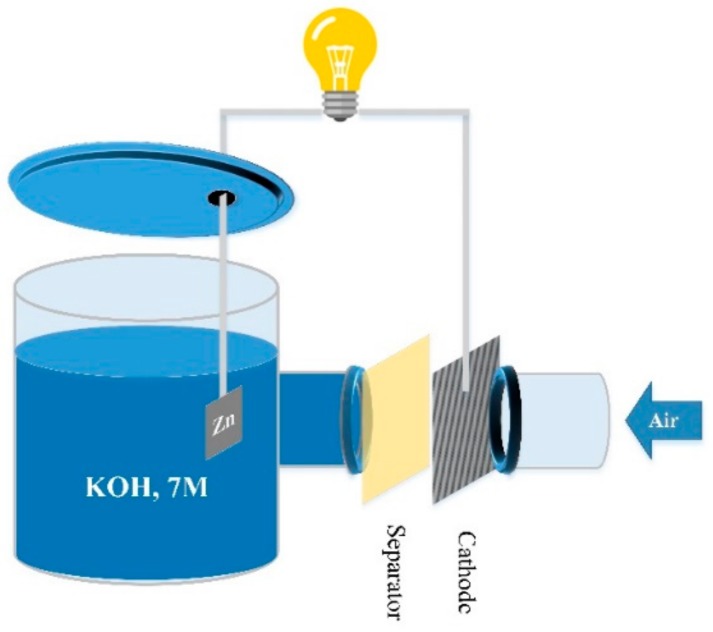
Schematic view of the discharge zinc–air cell.

A homemade zinc–air cell was used to evaluate the performance of the separator membranes. In this cell, the separator was in direct contact with the cathode, and the anode was a 1 × 1 cm^2^ pure zinc plate immersed in 40 mL of KOH, 7 M solution (Figure 12). To prepare the cathode, a Ni-foam was used as the current collector and gas diffusion layer. One side of the foam was coated with a mixture of BP-2000 (30%) and PTFE (70%), dispersed in ethanol and pressed using a hot-press at 350 °C for 15 min (air diffusion side). For preparing the catalyst side of the foam, a mixture of MnO_2_ (1.2 g: 30%), BP-2000 (1.4 g: 35%) and VXC-72 (1.4 g: 35%) was used. The mixture was stirred in 35 mL toluene for 2 h, and then 5 mL of 7.5 wt% poly(styrene-co-butadiene) solution in toluene (as a binder) was added and stirred for another 2 h. The final mixture was coated onto the Ni foam and pressed using a manual hot-press at 150 °C for 10 min. The size of the circular cathode used in the battery tests was 15 mm in the diameter.

Discharge performance was measured using a Battery Testing System (NEWARE, Shenzhen, China) at room temperature. The cell was discharged at a constant discharge current in the range of 2.5 to 15 mA/cm^2^. For all experiments, the cut-off voltage was 0.9 V.

## 4. Conclusions

Three PPO-based hydroxide exchange separator membranes, containing TMA, MPY, and MIM as quaternization agents, were developed. PPO–TMA and PPO–MPY exhibited excellent characteristics, required for rechargeable zinc–air batteries. They offered a decent ionic conductivity of ~0.17 mS/cm along with very low zincate diffusion coefficient of 1.13 × 10^−8^ and 0.28 × 10^−8^ cm^2^/min for PPO–TMA and PPO–MPY, respectively. Besides, their excellent chemical and thermal stability, and wide electrochemical stability window of higher than 3 V make them a suitable candidate separator for the batteries. Polarization characteristics of the batteries, using these membranes, showed improved discharge current density, high discharge capacity, and high discharge power. The results concluded that PPO–TMA and PPO–MPY significantly enhanced the performances of the batteries. Also, they represent a promising candidate separator for rechargeable zinc–air batteries.

## Figures and Tables

**Table 1:** Basic properties of PPO–TMA, PPO–MPy, and PPO–MIM separator membranes.

Sample	DI Water			KOH, 7 M			Ionic Conductivity (mS/cm)—σ	Zincate Diffusion Coefficient (×10^−8^ cm^2^/min)—D
	Uptake Δ*W* (wt%)	Area Change Δ*A* (%)	Volume Change Δ*V* (%)	Uptake Δ*W* (wt%)	Area Change Δ*A* (%)	Volume Change Δ*V* (%)		
PPO–TMA	89	65	119	31	11	39	0.17	1.13
PPO–MPy	78	41	76	30	11	39	0.16	0.28
PPO–MIM ^1^	13	14	30	3	0	0	0.003	N/A

^1^ Due to very low electrolyte uptake of PPO–MIM and its very low ionic conductivity, this membrane was not included in the rest of the study.

## Abbreviations

+FPS	Filter paper-bases separator
MIM	1-Methylimidazolium
MPY	1-Methylpyrolinine
TMA	Trimethylamine
PPO	Poly (2,6-dimethyl-1,4-phenylene oxide)
PPO–MIM	Quaternized PPO using 1-methylimidazolium
PPO–MPY	Quaternized PPO using 1-methylpyrolinine
PPO–TMA	Quaternized PPO using trimethylamine
