# Correction: Abbasi, A. et al. Poly(2,6-dimethyl-1,4-phenylene oxide)-Based Hydroxide Exchange Separator Membranes for Zinc-Air Battery. *Int. J. Mol. Sci.* 2019, *20*, 3678

**DOI:** 10.3390/ijms21020377

**Published:** 2020-01-07

**Authors:** Ali Abbasi, Soraya Hosseini, Anongnat Somwangthanaroj, Ahmad Azmin Mohamad, Soorathep Kheawhom

**Affiliations:** 1 Department of Chemical Engineering, Faculty of Engineering, Chulalongkorn University, Bangkok 10330, Thailand; abbasi.1000@gmail.com (A.A.); soraya20h@gmail.com (S.H.); anongnat.s@chula.ac.th (A.S.); 2 Computational Process Engineering Research Laboratory, Chulalongkorn University, Bangkok 10330, Thailand; 3 School of Materials and Mineral Resources Engineering, Universiti of Sains Malaysia, Nibong Tebal 14300, Pulau Pinang, Malaysia; aam@usm.my

The authors would like to make the following corrections to their paper published in the International Journal of Molecular Science [1]. The ionic conductivities shown in Table 1 were wrong because of the inconsistent unit of the thickness of membranes used in the calculation. In the corrected version, we updated the ionic conductivity and added the thickness, area, and bulk resistance of each membrane. The following changes are noted. The changes do not affect the conclusions of the article.

## 1. Change in Table 1

Table 1 should be replaced with the following:

## 2. Change of Figure 5

Figure 5 should be replaced with the following (the units of resistance in the insert image was changed to [Ω.cm^2^]):

## 3. Changes in Text

Lines 11–13 of the Abstract should be replaced with the following text:

Ionic conductivity of PPO–TMA, PPO–MPY, and PPO–MIM was determined using electrochemical impedance spectroscopy to be 17.37, 16.25, and 0.29 mS/cm, respectively.

Lines 1–11 on page 7 should be replaced with the following text:

Also, very low electrolyte uptake of PPO–MIM was reflected in the ionic conductivity measurements, showing very low conductivity of 0.29 mS/cm determined using a Nyquist plot of electrochemical impedance spectroscopy (EIS) (Figure 5). For PPO–TMA and PPO–MPy, the ionic conductivity was calculated to be 17.37 and 16.25 mS/cm. Due to deficient electrolyte uptake and low ionic conductivity of PPO–MIM, it was not included in the rest of the study.

Slightly higher ionic conductivities have been reported for the same separator membranes, which could be attributed to the higher measurement temperature and lower KOH solution concentration. In this study, the measurements were carried out in KOH, 7 M solution to mimic the real cell operation condition. As can be seen in Table 1, the separator membranes absorb much less electrolyte than they do in water, resulting in lower measured ionic conductivity.

Lines 3–5 in the Conclusion should be replaced with the following text:

They offered a good ionic conductivity of ~17 mS/cm along with very low zincate diffusion coefficient of 1.13 × 10^−8^ and 0.28 × 10^−8^ cm^2^/min for PPO–TMA and PPO–MPY, respectively.

We apologize for any inconvenience caused to the readers by this error.

## Figures and Tables

**Figure 5:**
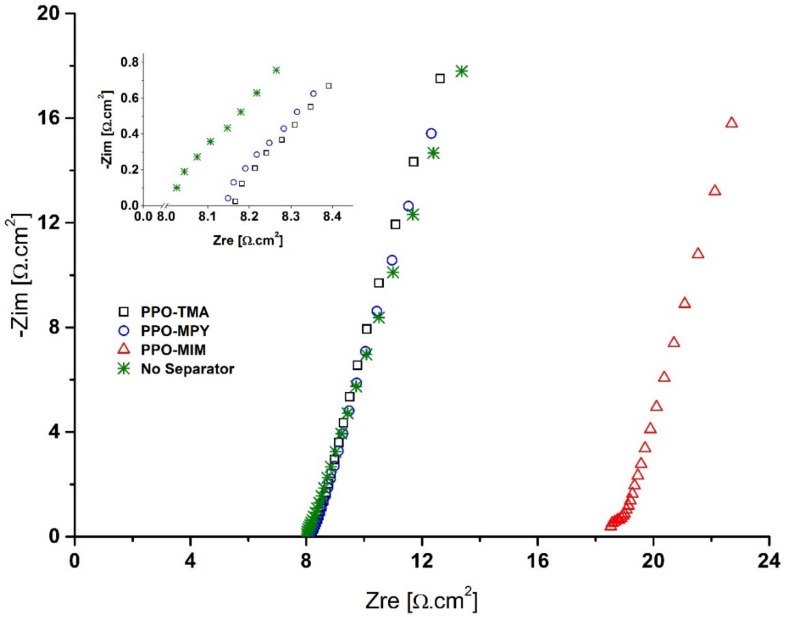
Nyquist plot of EIS for determining ionic conductivity of PPO-based separator membranes. The values of Zre and Zim were obtained by multiplying R_b_ values by the samples’ area (1.766 cm^2^).

**Table 1:** Basic properties of PPO-TMA, PPO-MPy, and PPO-MIM separator membranes.

Sample	DI Water			KOH, 7M			Thickness (μm)	Area (cm^2^)	R_b_ (Ω) ^2^	Ionic Conductivity (mS/cm)-σ	Zincate Diffusion Coefficient (× 10^−8^ cm^2^/min)-D
	Uptake ΔW (wt%)	Area Change ΔA (%)	Volume Change ΔV (%)	Uptake ΔW (wt%)	Area Change ΔA (%)	Volume Change ΔV (%)					
PPO-TMA	89	65	119	31	11	39	50	1.766	0.1630	17.37	1.13
PPO-MPy	78	41	76	30	11	39	40	1.766	0.1394	16.25	0.28
PPO-MIM ^1^	13	14	30	3	0	0	30	1.766	5.8014	0.29	N/A ^1^

^1^ Due to very low electrolyte uptake of PPO-MIM and its very low ionic conductivity, this membrane was not included in the rest of the study. ^2^ R_b_ values were obtained by deducting the value obtained for the cell without using any separator (4.4606 Ω) from the resistance values measured for each sample.

## References

[1] Abbasi A., Hosseini S., Somwangthanaroj A., Mohamad A.A., Kheawhom S. (2019). Poly(2,6-Dimethyl-1,4-Phenylene Oxide)-Based Hydroxide Exchange Separator Membranes for Zinc–Air Battery. Int. J. Mol. Sci..

